# Downregulation of CD147 expression alters cytoskeleton architecture and inhibits gelatinase production and SAPK pathway in human hepatocellular carcinoma cells

**DOI:** 10.1186/1756-9966-27-50

**Published:** 2008-10-11

**Authors:** Ai-Rong Qian, Wei Zhang, Jian-Ping Cao, Peng-Fei Yang, Xiang Gao, Zhe Wang, Hui-Yun Xu, Yuan-Yuan Weng, Peng Shang

**Affiliations:** 1 Key Laboratory for Space Biosciences & Biotechnology, Institute of Special Environmental Biophysics, Faculty of Life Sciences, Northwestern Polytechnical University Xi'an 710072, PR China

## Abstract

**Background:**

CD147 plays a critical role in the invasive and metastatic activity of hepatocellular carcinoma (HCC) cells by stimulating the surrounding fibroblasts to express matrix metalloproteinases (MMPs). Tumor cells adhesion to extracellular matrix (ECM) proteins is the first step to the tumor metastasis. MMPs degrade the ECM to promote tumor metastasis. The aim of this study is to investigate the effects of small interfering RNA (siRNA) against CD147 (si-CD147) on hepatocellular carcinoma cells' (SMMC-7721) architecture and functions.

**Methods:**

Flow cytometry and western blot assays were employed to detect the transfection efficiency of si-CD147. Confocal microscopy was used to determine the effects of si-CD147 on SMMC-7721 cells' cytoskeleton. Invasion assay, gelatin zymography and cell adhesion assay were employed to investigate the effects of si-CD147 on SMMC-7721 cells' invasion, gelatinase production and cell adhesive abilities. Western blot assay was utilized to detect the effects of si-CD147 on focal adhesion kinase (FAK), vinculiln and mitogen-activated protein kinase (MAPK) expression in SMMC-7721 cells.

**Results:**

Downregulation of CD147 gene induced the alteration of SMMC-7721 cell cytoskeleton including actin, microtubule and vimentin filaments, and inhibited gelatinase production and expression, cells invasion, FAK and vinculin expression. si-CD147 also blocked SMMC-7721 cells adhesion to collagen IV and phosphorylation level of SAPK/JNKs. SAPK/JNKs inhibitor SP600125 inhibited gelatinase production and expression.

**Conclusion:**

CD147 is required for normal tumor cell architecture and cell invasion. Downregulation of CD147 affects HCC cell structure and function. Moreover, the alteration of cell behavior may be related to SAPK/JNK Pathway. siRNA against CD147 may be a possible new approach for HCC gene therapy.

## Background

CD147 is a member of immunoglobulin (Ig) superfamily that takes an essential role in several normal tissues but is particularly enriched on the surface of malignant tumor cells *in vitro *and *in vivo *[1]. CD147 stimulates production of several matrix metalloproteinases (MMPs) by fibroblasts and endothelial cells [2,3]. Gelatinase, including MMP-2 and MMP-9, is one of the important MMPs and has been proposed to participate in human tumor invasion and metastasis. Other names for CD147 include human basigin, extracellular matrix metalloproteinase inducer (EMMPRIN), and human leukocyte activation-associated M6 antigen [4]. Homologues in other species include rat OX-47 antigen, mouse basigin or gp42 and chicken HT7 molecules [5]. CD147 has been thought to act outside the cell, primarily through MMPs, even though CD147 has also been shown to interact with several different molecules suggesting that at a molecular level it may be multifunctional [6,7]. Lim et al [8] found that EMMPRIN up-regulated MMP-1 mRNA expression, which was dependent on tyrosine kinase activity. Our previous studies have demonstrated that HAb18G/CD147 stimulates fibroblast cells to produce elevated levels of several MMPs, including MMP-1, MMP-2, and MMP-9, which are well known for prompting invasion of hepatocellular carcinoma (HCC) cells and antisense RNA of HAb18G/CD147 inhibited the invasion of HCC cells in vitro [9]. The expression of HAb18G/CD147 reduced the sensitivity of the store-operated Ca^2+ ^entry to NO/cGMP and enhanced the metastatic potentials of HCC cells [10]. Our previous work has identified 9 binding peptides by screening a random 12-mer phage peptide library. The roles of these peptides inhibiting HCC cell invasion, adhesion and angiogenesis were analyzed [11-13].

Metastasis formation is a multistep process that requires tumor cells to progress through many different stages [14]. Proteolysis of the extracellular matrix (ECM), as well as increased locomotion, leads to intravasation and dissemination of tumor cells. Experimental evidences have shown that cancer cells are armed with an array of proteolytic enzymes that appear to be essential for the process of cancer dissemination [15]. Tumor cell adhesion, deformability, motility, and cell receptors also play important roles in cancer invasion.

In all mammalian cells, the cytoskeleton is represented by three types of filamentous structures, including actin microfilaments (AFs), intermediate filaments (IFs), and microtubules (MFs). The cytoskeleton is a dynamic cell's internal filamentous network whose formation and remodeling underlies the fundamental processes of cell motility and shape determination. The cytoskeleton not only has function in maintaining cell shape, also involves in cell motility and mitosis [16]. Tumor cell motility is a critical step in tumor invasion and metastasis. In particular, it is known that membrane-associated cytoskeletal proteins are required for tumor cell movement and infiltration to surrounding tissue [17].

Vinculin, which is one kind of cytoskeletal proteins involved in formation of the cytoplasmic scaffolding, takes an important role in cell adhesion and migration by providing the link between the actin cytoskeleton and the transmembrane receptors, integrin and cadherin [18]. Vinculin can regulate the ability of cancer cells to move away from tumors and spread cancer to other parts of the body [19]. FAK is a critical regulator of cell adhesion and migration and active FAK can recruit additional structural and signaling molecules to contribute to the assembly of focal adhesion complex [20,21].

The mitogen-activated protein kinase (MAPK) superfamily is composed of three major sets of kinases, namely, the extracellular-receptor kinases (ERK), the stress-activated protein kinases/c-Jun N-terminal kinases (SAPK/JNKs) and the p38 MAPKinases [22]. SAPK/JNKs are members of the MAPK family and are activated by a variety of environmental stresses. SAPK signaling cascade is involved in the regulation of many cellular processes and the research has recently demonstrated a functional role for SAPK in the suppression of metastases [23].

RNA interference (RNAi) technology has been rapidly adopted for the discovery and validation of gene function through the use of a sequence-specific short interfering RNA (siRNA), which was originally identified as 21–23-nucleotide double-stranded RNA (dsRNA) fragments, generated by a cellular enzyme called Dicer [24]. The use of RNAi is spreading rapidly to nearly every aspect of biomedical research. The gene silencing capability of RNAi is being used to study individual gene's biological function and role in biochemical pathways.

Our previous studies have showed that siRNA of CD147 inhibited HAb18G/CD147 expression in HCC cells by RT-PCR and the inhibitory rate was more than 70% (the results have been submitted). Recently, our results showed that dsRNA targeted for CD147 inhibits MMP-2 secretion and FAK expression in HCC cell line FHCC98 [25]. The results of microarray results showed that downregulation of CD147 leads to some cytoskeletal genes alteration (the results have been submitted).

Although many studies on CD147 functions have been widely carried out, the mechanism of tumor metastasis induced by CD147 is still not clear, especially in HCC. In order to further explore the mechanism, we employed RNAi method to study the effects of si-CD147 on HCC cell cytoskeleton, cell invasion, cell adhesion, the expression of vinculin, FAK and MAPK. Our data indicated that si-CD147 inhibited HCC cells' invasion, adhesion, gelatinase production and induced cytoskeleton alteration.

## Methods

### Cell Culture

Human HCC cell line SMMC-7721 was obtained from China Centre for Type Culture Collection, Chinese Academy of Sciences. SMMC-7721 cells were grown in complete RPMI 1640 culture medium (GIBCO™ Invitrogen Corporation, Carlsbad, California) supplemented with 10% fetal calf serum (Hyclone, Utah, USA), 1% penicillin/streptomycin, and 2% L-glutamine at 37°C in a humidified atmosphere of 5% CO_2_.

### siRNA synthesis and transfection

SiRNA designed to target CD147 (Sense Sequence: GGUUCUUCGUGA GUUCCUCtt, Antisense Sequence: GAGGAACUCACGA AGAACCtg) was synthesized by Ambion Research Inc. As a negative control, we used stealth™ RNAi negative control Hi-GC (12935-400) (Invitrogen Corporation, Carlsbad, California). SMMC-7721 cells were trypsinized and added into 6-well dishes (Nunc Inc in Denmark) (6 × 10^5 ^cells/well). Cells were cultured with growth medium without antibiotics until the 90% confluent at the time of transfection. 5 μl siRNA (50 nmol/ml) of CD147 and 6 μl lipofectAMINE 2000 (Invitrogen Corporation, Carlsbad, California) was respectively added to 500 μl RMPI-1640 medium without serum. After the 5 min incubation, the diluted siRNA (250 pmol) of CD147 and lipofectamine 2000 were mixed gently and incubated for 20 min at room temperature (RT) to allow complex formation to occur. The RNAi-lipofectAMINE 2000 complexes were added to each well containing cells and mixed gently. The cells were incubated at 37°C in a humidified atmosphere of 5% CO_2 _for 48 h.

### Flow cytometric analysis of CD147 expression

After being transfected with si-CD147 or control siRNA for 48 h, SMMC-7721 cells were collected and labeled with anti-CD147 (BD Pharmingen, USA) for 1 h at RT. Cells were washed three times and co-cultured with goat anti-mouse IgG-FITC (1:100, KPL, Oklahoma, USA) at 4°C in dark for 30 min and subjected to flow cytometric analysis using a FACSCalibur flow cytometer (BD Biosciences, California, USA) and CellQuest software (BD Biosciences, California, USA).

### Western blot analysis of CD147, FAK, vinculin, MMP-2, MMP-9 Erk1/2, p38, and SAPK/JNK MAP kinases

SMMC-7721 cells (1.0 × 10^5^/ml) transfected with si-CD147 or control siRNA suspension were collected, and centrifuged at 800 r/min. The cell pellets were collected and resuspended in lysis buffer of 50 mmol/l Tris-HCl (pH 8.0), 150 mmol/l NaCl, 0.02% azido sodium, 0.1% SDS, 100 μg/ml PMSF (phenylmethyl sulfonylfluoride), 1% Triton X-100, 0.5% deoxycholic acid and protease inhibitor for 10 min on the ice. The lysis solution was centrifuged at 16000 g for 10 min at 4°C and the supernatants were collected, and the protein concentration was detected with Lowery's method. Equal total protein loads of 100 μg were loaded on the lanes of SDS-polyacrylamide gel and separated by electrophoresis in a 5% acrylamide stacking gel and an 8% acrylamide separating gel. Protein marker as the molecular weight ladders was loaded at the same time. The proteins then transferred to PVDF membrane (Immobilon-P, Millipore, Bedford, MA, USA). After being blocked with 5% (w/v) Odyssey blocking buffer (Li-COR Bioscience), the blots were incubated for overnight with antibodies directed against CD147 (BD Pharmingen), FAK(Sigma Aldrich, America) and vinculin (Merck Calbiochem) at 4°C. Blots were washed 5 min three times with tris-buffered saline/0.1% Tween-20 and incubated with IRDye™ 800-labeled secondary antibodies (Li-COR Biosciences, Nebraska, USA) (1:5000) for 1 h and identical lysates were reprobed with a GAPDH antibody (Chemicon International, Inc., California, USA) as a loading control. The membranes were washed as above, and the bands were scanned by Odyssey Infrared Imaging System (LI-COR Biosciences, Nebraska, USA). The expressions of MMP-2, MMP-9, MAPK expression in SMMC-7721 cells transfected with CD147 siRNA, and the effects of SAPK/JNK inhibitor SP600125 (Beyotime Biotech, China) on MMP-2 and MMP-9 expression in SMMC-7721 cells were also detected by using chemiluminescence (Santa Cruz Biotechnology Inc) after 30 s-1 min exposures. MAPK family antibody sampler kit and phospho-MAPK family antibody sampler kit were purchased from Cell Signaling Technology, and anti-MMP-2 and anti-MMP-9 were from Merck Calbiochem.

### Gelatin Zymography analysis of gelatinase production

After being transfected with si-CD147 or control siRNA for 48 h, SMMC-7721 cells were continuously cultured for 15 h in serum-free RPMI 1640. The supernatants were collected and centrifuged to remove debris. Conditioned medium were separated by SDS-PAGE under nonreducing conditions using 8% separating gel containing 0.1% gelatin. The gels were incubated in 2.5% Triton X-100 solution at room temperature with gentle agitation to remove SDS and were soaked in reaction buffer 50 mM Tris-HCl (pH 7.5), 200 mM NaCl, 10 mM CaCl_2_, at 37°C overnight. After reaction, the gels were stained for 6 h with staining solution and were destained about 0.5 h. Gelatinolytic activity of MMPs was visualized as a clear band against a dark background of stained gelatin. The MMP-2 and MMP-9 production were also investigated after SMMC-7721 cells were treated with SP600125 (100 μM) for 1 h by gelatin zymography.

### Invasion assay

*In vitro *invasion assay was performed by using millicell inserts (Millipore Corp) with polycarbonate filters (pore size, 8 μm). The upper side of polycarbonate filter was coated with matrigel (0.6 mg/ml, BD Biosciences) to form a continuous thin layer. Prior to the addition of cell suspension, the dried layer of matrigel matrix was rehydrated with medium without serum for 2 h at RT. The Cells transfected with si-CD147 or control siRNA were harvested and 2.2 × 10^5 ^cells in 300 μl of 0.1% serum-medium were placed in the upper chamber. The lower chamber was filled with 0.1% serum-medium (200 μl) and serum-free conditioned medium from NIH/3T3 (300 μl). Cells were cultured for 24 h at 37°C in 5% CO_2_. After incubation, the cells were washed three times with RPMI and were then fixed by immersing the chambers in 95% methanol for 10 mins. Cells on the upper surface were removed with a cotton swab and were stained with hematoxylin and eosin. Cells migrated on the underside of the membrane were counted microscopically. The determinations were done in triplicate.

### Cell adhesion assay

SMMC-7721 cells (5 × 10^4^) transfected with si-CD147 or control siRNA were suspended and added in 96-well micro-plates coated with fibronectin (FN), laminin (LN), and collagen IV (3 μg per well, BD Biosciences). The cells were allowed to adhere for 60 min at 37°C. The wells were washed three times with PBS and the cells were fixed with 4% formaldehyde. The cells were then stained with 0.5% crystal violet in 20% (v/v) methanol/water and viewed under a microscope. The amount of bound cells was estimated by solubilizing the dye using 0.1 M sodium citrate and reading the absorbance at 490 nm. Triplicate determinations were done at each data point.

### Confocal microscopy

Cells transfected si-CD147 or control siRNA for 48 h were collected and added to the sterile coverslips and cultured overnight. The cells were washed twice with pre-warmed phosphate-buffered saline (PBS), pH 7.4. For antibodies that have unknown properties on fixed cells it is best to start with one fixing condition that preserves native structure, so we chose different fixative dependent on different aims. We chose -20°C methanol for 1–2.5 minutes to fix cells for actin filaments. 0.5% glutaraldehyde fixation was chose for tubulin and vimentin filaments. Then the fixed cells were washed with TBS and blocked with 2% BSA in TBS-0.1% TX for 10 mins. Anti-actin (Sigma Aldrich, America), Anti-tubulin (Merck Calbiochem) and anti-vimentin (Merck Calbiochem) were added to the fixed cells for 4°C overnight and then sheep anti mouse FITC-IgG (1:100, KPL, Oklahoma, USA) was added for 60 mins at RT in dark. The cells were washed for three times with TBS, enveloped with glycerol, and then observed by confocal microscope (Leika DMIRE2/TCS SP2).

### Statistical analysis

Statistically significant differences were determined Prism statistical soft (GraphPad Software). *P *< 0.05 was considered significant in all cases.

## Results

### SiRNA against CD147 inhibits CD147 expression in SMMC-7721 cells

**Figure 1:**
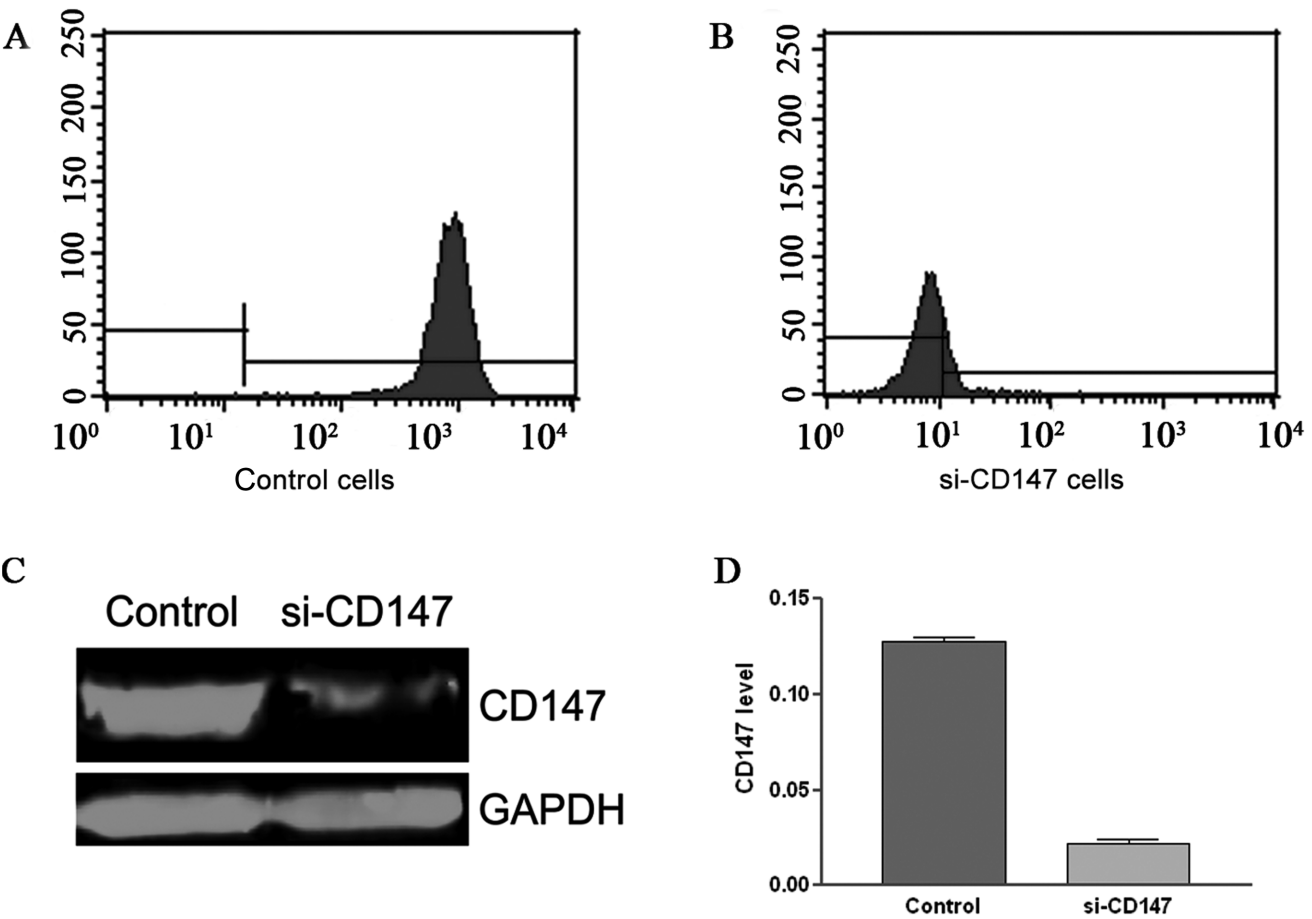
**CD147 expression in SMMC-7721 cells transfected with negative control siRNA or siRNA-CD147**. After SMMC-7721 being transfected with negative control siRNA or si-CD147 for 48 h, CD147 expression was detected using flow cytometry (A, B) and western blot assay(C, D). GAPDH as a loading control was shown in the bottom panel. The PVDF membranes were washed and the bands were scanned by Odyssey Infrared Imaging System (C). The graph (D) compares scanning signal intensity of CD147 by ImageJ software.

Our previous results suggested that CD147 was highly expressed in HCC cells [9]. To analyze whether siRNA against CD147 could specifically inhibited CD147 expression, we used flow cytometric analysis and western blot assay to determine the effects of si-CD147 on CD147 expression in SMMC-7721 cells. The results showed that the expression of CD147 in SMMC-7721 cells transfected with si-CD147 was significantly decreased with an inhibitory rate over 80% compared with that in negative control cells (Figure. 1).

### Downregulation of CD147 inhibits MMP-2 and MMP-9 expression in SMMC-7721 cells

**Figure 2:**
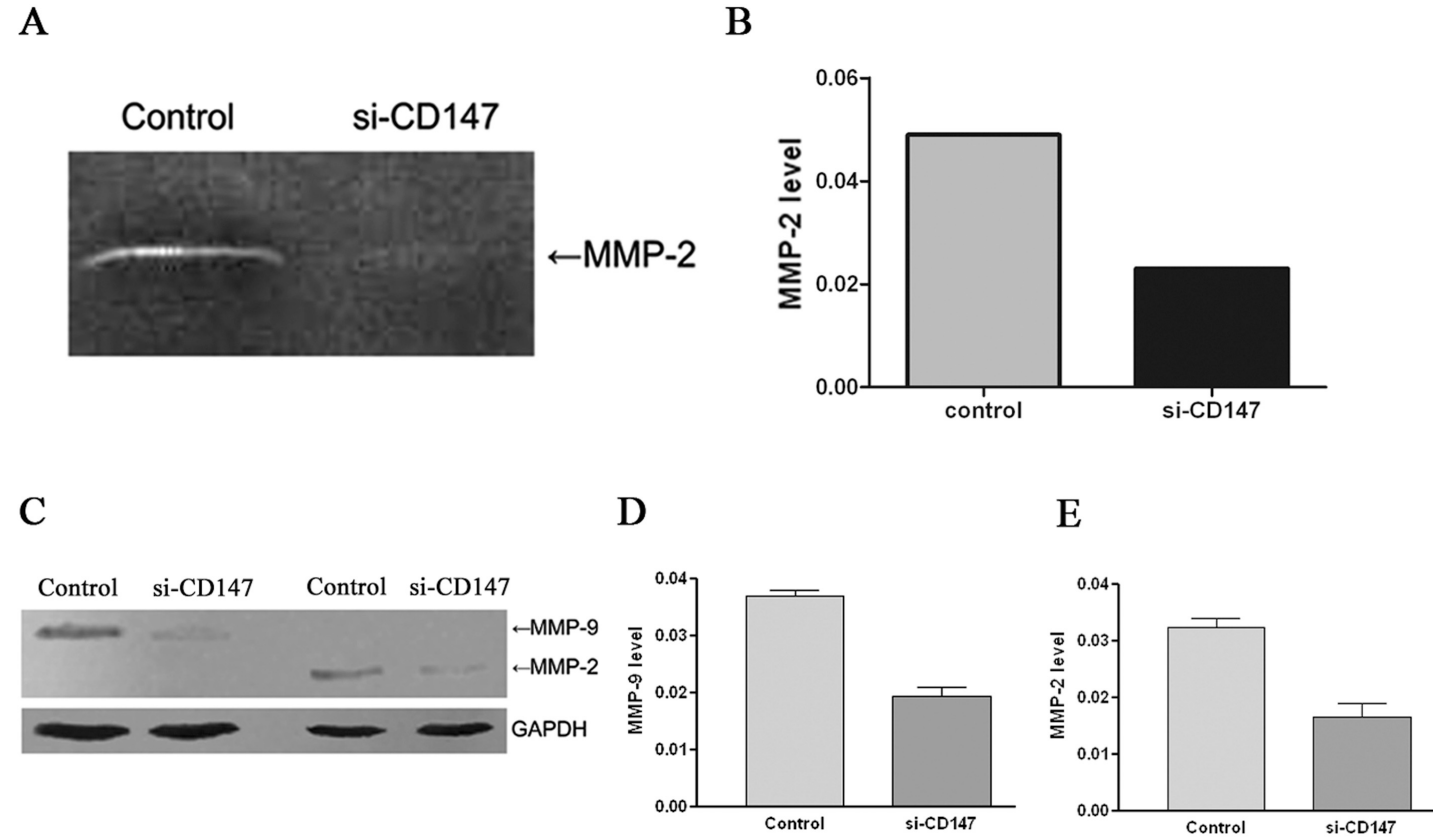
**Detection of the effects of si-CD147 on gelatinase production by gelatin zymography (A, B) and western blot assay (C, D, E)**. The production and expression of gelatinase were detected by gelatin zymography (A, B) and western blot assay (C, D, E), after SMMC-7721 cells were treated with si-CD147 or negative control siRNA for 48 h. MMP-2 production was decreased significantly in SMMC-7721 cells transfected with si-CD147 (A). The graph (B) compares scanning signal intensity of MMP-2 by ImageJ software. MMP-2 and MMP-9 expression greatly decreased in SMMC-7721 cells transfected with si-CD147 (C). The graph (D, E) compares scanning signal intensity of MMP-9 and MMP-2 expression by ImageJ software.

To investigate whether the downregulation of CD147 in SMMC-7721 affects MMPs secretion, we evaluated MMP-2 and MMP-9 expression in SMMC-7721 cells transfected with si-CD147 by gelatin zymography and western blot assay. Gelatin zymograpy analysis showed that MMP-2 production in SMMC-7721 cells transfected with si-CD147 was decreased by over 50% compared with that in negative control cells (Figure. 2A and 2B). Western blot analysis showed that both of the MMP-2 and MMP-9 expression were inhibited in SMMC-7721 cells transfected with si-CD147 compared with that in control cells (Figure. 2C, 2D and 2E).

### Downregulation of CD147 gene blocks SMMC-7721 cells' invasion

**Figure 3:**
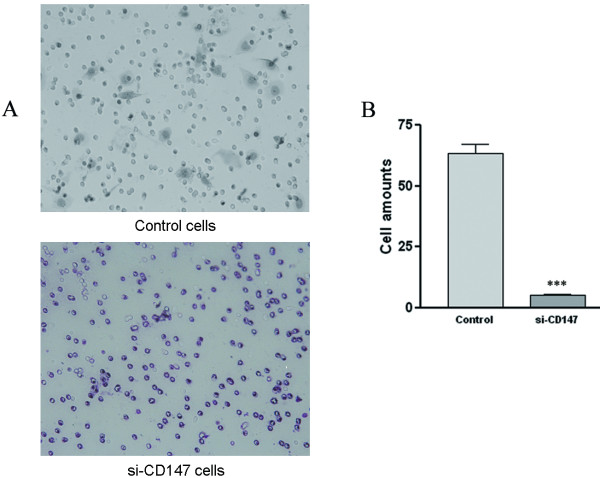
**Effects of si-CD147 RNA on SMMC-7721 cells' invasive abilities**. The invasive abilities of SMMC-7721 cells transfected with negative control and si-CD147 RNA were examined by chamber assay. HE staining and quantitative analysis were employed to analyze the number of the transmembrane cells. Transmembrane cells number dramatically reduced in SMMC-7721 cells transfected with si-CD147 (A). The graph (B) compares the number of transmembrane cells and indicates an 85% inhibition by si-CD147. ***P < 0.001 *v.s. *control.

In order to confirm whether CD147 is involved in the process of cell motility, we used invasion assay to determine the impact of siRNA-CD147 on SMMC-7721 cells' invasion. 5 × 10^5 ^cells transfected with si-CD147 or control siRNA were added to millicell inserts coated with matrigel. We observed that the number of infiltrating cells was significantly decreased in SMMC-7721 cells transfected with si-CD147 compared with that in control cells and the inhibitory rate was about 85% (Figure. 3A and 3B).

### Downregulation of CD147 inhibited SMMC-7721 cells adhesion to collagen IV

**Figure 4:**
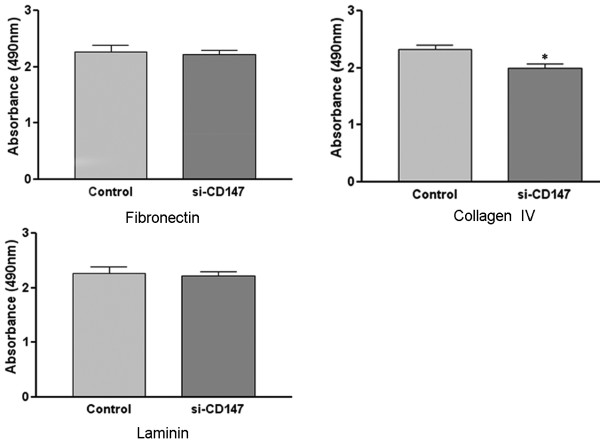
**Effects of si-CD147RNA on SMMC-7721 cells adhesion to fibronectin, collagen IV and laminin**. SMMC-7721 cells (5 × 10^4^) transfected with negative control or si-CD147 were added to 96-well micro-plates coated with FN, LN, and collagen IV. The cells were allowed to adhere for 60 min at 37°C and were then stained with 0.5% crystal violet in 20% (v/v) methanol/water. The amount of bound cells was estimated by solubilizing the dye using 0.1 M sodium citrate and reading the absorbance at 490 nm. The results indicated that si-CD147 inhibited SMMC-7721 adhesion to collagen IV but had no effects on adhesion to FN and LN. *P < 0.05 *v.s. *control.

The effects of si-CD147 on the adhesive abilities of HCC cells to ECM proteins including FN, LN and collagne IV were detected by adhesion assays. The results indicated that si-CD147 inhibited SMMC-7721 adhesion to collagen IV but had no effects on SMMC-7721 cells adhesion to FN and LN (Figure. 4).

### Downregulation of CD147 results in the cytoskeletal alteration of SMMC-7721 cells

**Figure 5:**
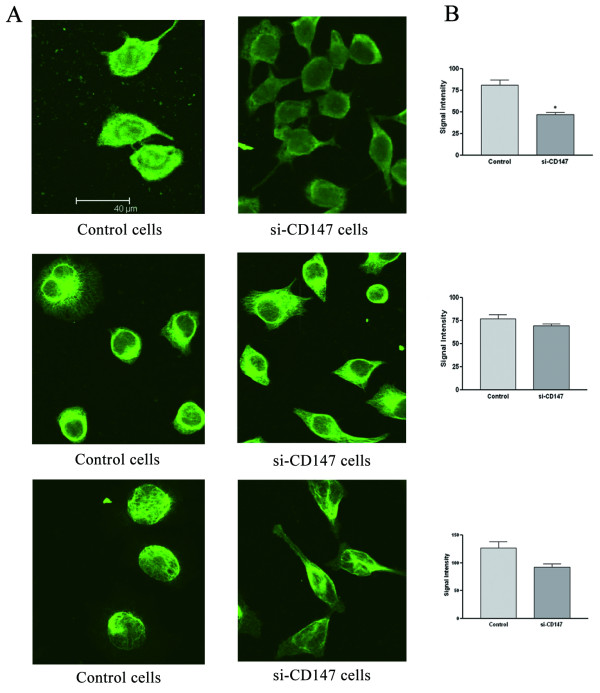
**The effects of si-CD147 on SMMC-7721 microfilament actin, microtubule and vimentin**. After being transfected with negative control or si-CD147 for 48 h, cells were trypsinized and cultured on coverslips, and then fixed. The effects of si-CD147 on SMMC-7721 cytoskeleton were investigated by confocal microscopy. Actin, mirotublin and vimentin filaments in si-CD147 transfected cells were significantly altered (A) and the mean signal intensities were quantified with ImageJ software (B). *P < 0.05 *v.s. *control.

We examined the effects of siRNA-mediated CD147 gene silencing on SMMC-7721 cytoskeleton including actin filament, microtubule and intermediate filament by confocal microscopy. SMMC-7721 cells transfected with si-CD147 or control siRNA were labeled respectively with antibodies targeting actin, tublulin and vimentin to visualize these protein. In SMMC-7721 cells transfected with si-CD147, actin showed a nuclear concentration and decreased fluorescence intensity. (Figure. 5). The microtubule constructed to a network and cells appeared flattened in si-CD147 transfected cells, but the difference of fluorescence intensity between two groups was not significant (Figure. 5). Vimentin filaments showed an interlaced pattern in control cells but in transfected cells they seemed to form foci in the periphery of cells, and the difference of fluorescence intensity between two groups was not significant (Figure. 5).

### Downregulation of CD147 inhibits FAK and Vinculin expression in SMMC-7721 cells

**Figure 6:**
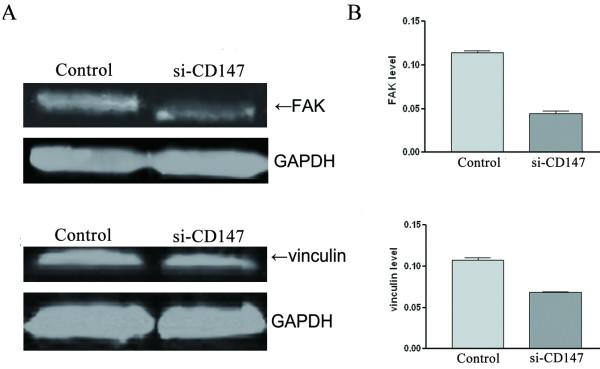
**Effects of si-CD147 on FAK and vinculin expression in SMMC-7721 cells**. Total cellular protein from SMMC-7721 cells tranfected with siRNA was analyzed by 10% SDS-PAGE and transferred to PVDF membrane prior to immunoblotting. The PVDF membranes were washed and the bands were scanned by Odyssey Infrared Imaging System. The results showed that si-CD147 inhibited FAK and vinculiln expression in SMMC-7721 cells (A). The graph (B) compares scanning signal intensity of FAK and vinculin expression by ImageJ software.

In order to further investigate whether CD147 is involved in signal transduction, we tested FAK and vinculin expression in SMMC-7721 cells transfected with si-CD147 by western blot. The results showed that si-CD47 significantly inhibited vinculin and FAK expression (Figure. 6A and 6B).

### siRNA of CD147 inhibits phosphorylation of SAPK/JNK but not other MAP kinases

**Figure 7:**
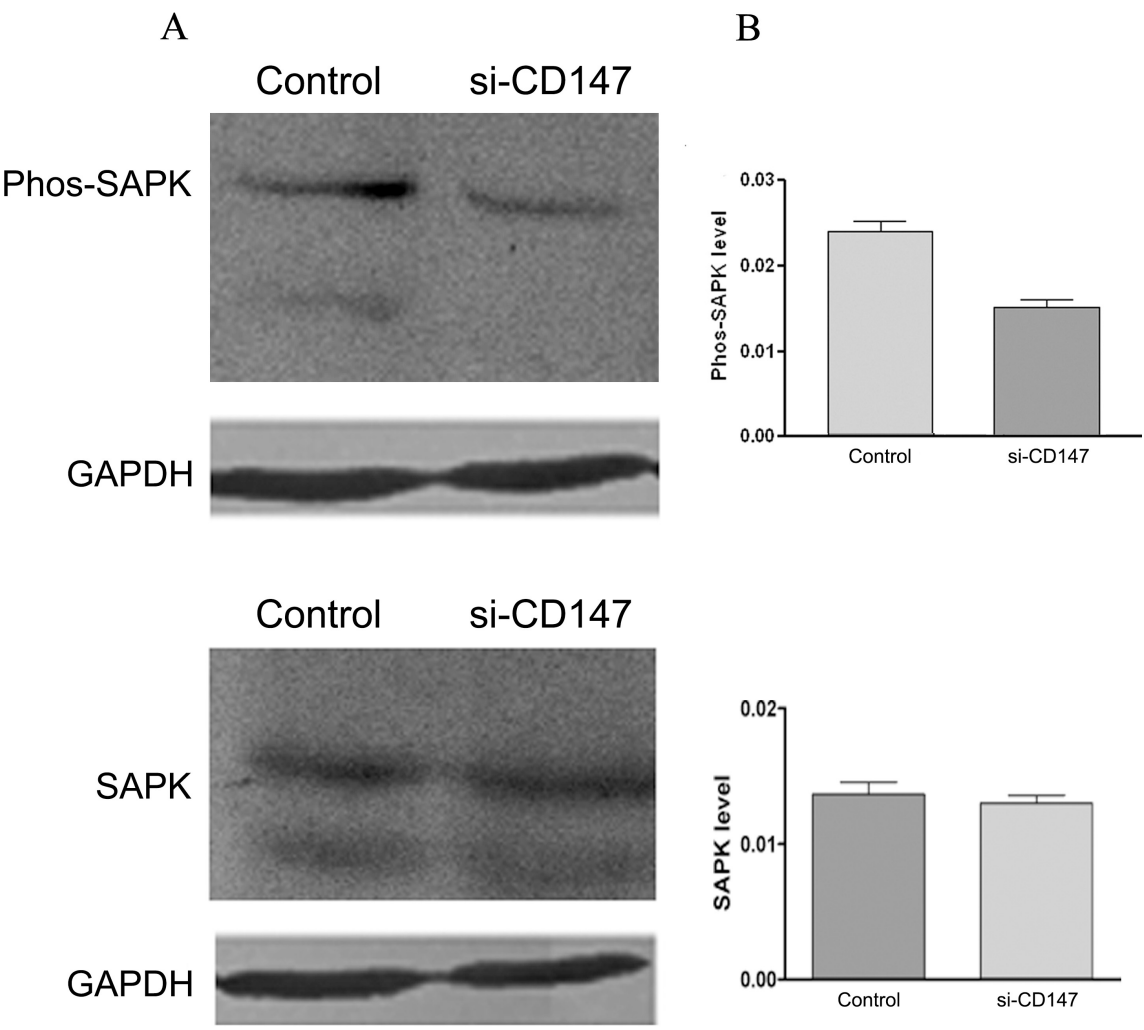
**Effects of si-CD147 on MAPK expression in SMMC-7721 cells**. p38, ERK1/2, and JNK MAP kinase expression levels were analyzed in SMMC-7721 cells, respectively. Western blot analysis showed that si-CD147 inhibited the phosphorylation level of SAP/JNK but had no effects on non-phosphorylation level of SAP/JNK (A). GAPDH (lower panel) served as a loading control. The graph (B) compares scanning signal intensity of phos-SAPK and non-phos-SAPK expression by ImageJ software.

MAPK pathway plays an important role in regulating MMPs production, cell motility and invasion, so we detect the effects of si-CD147 on MAPK. The results showed that si-CD147 inhibited phosphorylation of SAPK/JNK. No decreased signal was detected with the phospho-Erk/p44/p42 and phospho-ERK1/2 antibodies (Figure. 7). In addition, si-CD147 had no effects on nonphophorylation of MAPK (Figure. 7).

### SAPK/JNK inhibitor SP600125 blocks gelatinase production and expression

**Figure 8:**
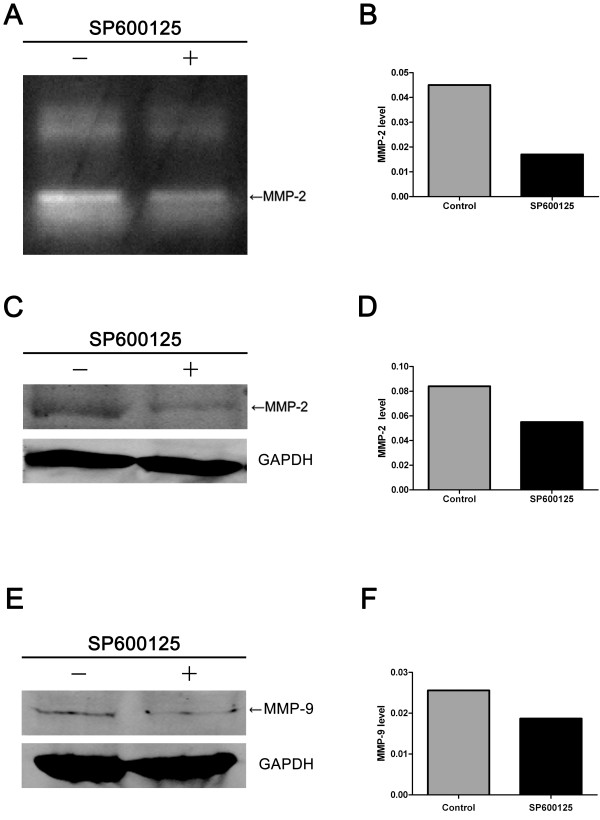
**Effects of SAPK/JNK inhibitor SP600125 on gelatinase production and expression**. Total cellular protein from SMMC-7721 cells treated with SP600125(100 μM) for 1 h was analyzed by gelatin zymography and western blot assay. The results showed that MMP-2 production (A) and expression (C), and MMP-9 expression (E) in SMMC-7721 cells treated with SP600125 was significantly inhibited. The graph (B, D, F) compares scanning signal intensity of MMP-2 and MMP-9 production and expression by ImageJ software.

In order to identify whether the production and expression of MMP-2 and MMP-9 are related to SAPK/JNK, we examined the effects of SAPK/JNK inhibitor SP600125 on MMP-2 an MMP-9 by gelatin zymography and western blot assay. The results showed that MMP-2 production was significantly reduced in SMMC-7721 cells treated by SP600125 (Figure 8A and 8B). SAPK/JNK inhibitor SP600125 also inhibited the expression of MMP-2 and MMP-9 in SMMC-7721 cells (Figure. 8C–F).

## Discussion

The primary mechanism of cell motility is related to reorganization of the actin cytoskeleton. Thus, a potentiality of regulating tumor cell invasion and metastasis by controlling actin cytoskeleton to control cell migration has been provided. Although there are many factors to regulate cell motility, the bundling and subcellular distribution of the actin cytoskeleton directly determines cell motility [26].

In this study the siRNA targeted CD147 was designed and synthesized and the effects of si-CD147 on SMMC-7721 cell's invasive abilities and cytoskeleton were investigated. Our findings showed that si-CD147 induced SMMC-7721 cytoskeleton alteration including actin, tubulin and viment filaments. Recently, in our lab the results of microarray results showed that downregulation of CD147 leads to some cytoskeletal genes alteration, such as vimentin gene is upreglated more than twice in hepatocellar carcinoma cells transfected si-CD147 (data not shown). Tsai WC [27] showed that actin and vimentin expression in breast phyllodes tumors correlates with tumor grades of the WHO grading system. Recent reports have reported the expression of vimentin in numerous neoplasms, including melanoma, which has been linked to metastatic disease [28]. It has been reported by the University of Iowa Health Care research group and by others that the co-expression of vimentin along with keratins in human melanoma is associated with recurrent and metastatic disease [29]. Khunkeawla et al [30] studied that U937 cell aggregation induced by CD147 mAbs is associated with cytoskeleton reorganization and the results showed that cell aggregation induced by the engagement of CD147 with specific mAbs depended upon the activation of protein kinases and a functional cytoskeleton. Together with the recent findings that antibodies to CD147 also neutralize cell aggregation mediated by CD98 [31], it may be speculated that CD147 plays a central role in cellular events associated with cytoskeleton rearrangement. Curtin et al [32] showed that D-basigin is necessary for cell to maintain normal architecture. D-basigin (EMMPRIN/CD147) interacts with integrin to affect cellular architecture and D-basigin partially colocalizes with actin in High Five cells. Schlosshauer, et al [33] was also previously reported that D-basigin partially colocalizes with actin in chick retinal pigment epithelium.

Tumor cell invasion and adhesion are very critical steps in tumor metastasis. In this study, the invasive abilities of SMMC-7721 cells were inhibited by si-CD147. The results further confirm that CD147 plays critical roles in tumor cell invasion. Wang, et al [34] showed that downregulation of CD147 by RNAi decreased the invasive capability of prostate cancer cells. Tumor cell metastasis involves a complex series of interdependent events, including repeated invasion of basement membranes (BM) [35]. The major component of the BM is known to be collagen IV. The results showed that si-CD147 inhibited SMMC-7721 cells adhesion to collagen IV but not FN and LN. This suggests that si-CD147 may inhibit specific receptors of collagen IV. Two specific receptor molecules of collagen IV, α1β1 and α2β1 have been identified. Integrin α1β1 and α2β1 are the key regulators of hepatocarcinoma cell invasion across the fibrotic matrix microenvironment [36].

Vinculin is a highly conserved cytoskeletal protein that is essential for regulation of cell morphology and migration, and is a critical component of both cell-cell and cell-matrix complexes [37]. The results of this study showed that si-CD147 inhibited vinculin expression. It indicates the decrease of vinculin expression maybe affects both cell-cell and cell-matrix complexes formation and finally influences SMMC-7721 cell motility and adhesion. Kuroda, *et al*, has reported that vinculin may serve as a useful marker of renal neoplasms with collecting duct system phenotype such as chromophobe-type renal cell carcinomas [38].

Focal adhesion formation is an essential step in cell adhesion [39]. Our data also showed that si-CD147 inhibited FAK expression in SMMC-7721. A number of reports have showed that Elevated FAK expression has been linked to the increased invasive potential of human tumors [40,41]. Lu, *et al *has reported that downregulation of FAK activity might be essential and required for early metastatic spreading, enabling vascular circulation of tumor cells without adhesion. Once tumor cells reattach to the ECM, integrin stimulation of FAK promotes adhesion and the growth of a metastatic tumor [42].

SAPKs/JNKs function in a protein kinase cascade transducing cellular stress signals and are activated by a highly diverse group of extracellular signals [43]. Simon, et al [44] showed that SAPK pathway regulates the expression of the MMP-9 collagenase via AP-1-dependent promoter activation. Our results showed that siRNA of CD147 inhibited phosphorylation of SAPK/JNK. It suggests that tyrosine phosphorylation-dependent MAP kinases SAPK/JNK but not ERK 1/2 and p38 kinases control the response to CD147. Moreover, SAPK/JNK specific inhibitor SP600125 blocked gelatinase expression and production, so it indicated that SAPK/JNK maybe participated in regulating gelatinase production.

Lim, et al [8] studied that EMMPRIN-mediated stimulation of MMP-1 synthesis in human lung fibroblasts is dependent on the activity of the MAP kinase, p38, but not ERK1/2 or SAPK/JNK. Si-CD147 inhibits phosphorylation of SAPK/JNK, which further confirms that gelatinase expression induced by CD147 depends on SAPK/JNK pathway. Our previous findings indicated that siRNA targeted against HAb18G/CD147 inhibits ERK1/2 pathway in HCC cell line FHCC98 [25]. The disparity may be related to different siRNA sequence and different cell lines, and the further mechanism is under study.

## Conclusion

CD147 is required for normal tumor cell architecture and cell invasion. siRNA targeted to CD147 inhibited HCC cell invasion, adhesion to Collagen IV and gelatinase production, and affected cytoskeleton structure. Moreover, the inhibition of gelatinase production may be related to the downregulation of phosphorylation-dependent SAPK/JNK.

## List of abbreviations

MMPs: matrix metalloproteinase; ECM: extracellular matrix; dsRNA: double-stranded RNA; siRNA: small interfering RNA; si-CD147: siRNA against CD147; HCC: hepatocellular carcinoma; FN: fibronectin; LN: laminin; EMMPRIN: extracellular matrix metallproteinase inducer; FAK: focal adhesion kinase; AFs: actin microfilaments; MFs: microtubules filaments; IFs: intermediate filaments; BM: basement membranes; FAK: focal adhesion kinase; GAPDH: glyceraldehyde 3-phosphate dehydrogenase; MAPK: mitogen-activated protein kinase; ERK: extracellular-receptor kinases; SAPK/JNKs: stress-activated protein kinases/c-Jun N-terminal kinases.

## Competing interests

My coauthors and I have no financial or other conflicts of interest that might influence the results or interpretation of our study.

## Authors' contributions

ARQ and PS carried out the design of the study, performed the statistical analysis and drafted the manuscript. WZ, ZW, PFY and HYX carried out the immunoassays and confocal microscope. JPC, YYW and XG participated in the cell culture, invasive assays and gelatin zymography. All authors read and approved the final manuscript.
